# Crash-Test Curve Anomaly Detection via Multi-View Context Augmentation

**DOI:** 10.3390/s26113298

**Published:** 2026-05-22

**Authors:** Chang Zhou, Boqin Zhang, Zhao Liu, Ping Zhu

**Affiliations:** 1 School of Mechanical Engineering, Shanghai Jiao Tong University, Shanghai 200240, China; tutbrother@sjtu.edu.cn (C.Z.);; 2 National Engineering Research Center of Automotive Power and Intelligent Control, Shanghai Jiao Tong University, Shanghai 200240, China; 3 School of Design, Shanghai Jiao Tong University, Shanghai 200240, China

**Keywords:** crash-test curves, crashworthiness assessment, data quality control, anomaly detection, multi-view context augmentation

## Abstract

In automotive crash testing, trustworthy crash-test curves are essential for reliable crashworthiness assessment, yet automated anomaly detection is difficult due to limited labeled abnormal cases, event-level data scarcity, and distribution shifts across vehicle models and sensor configurations. This paper proposes MVCA-AD (Multi-View Context Augmentation for Anomaly Detection) for single-channel crash-test curves. MVCA-AD generates multiple context-rich views using deterministic time- and frequency-domain transformations to amplify subtle anomalous patterns under limited labeled supervision. A trend-aware modulation module and cross-view attention fuse these views to improve sensitivity to critical segments such as impact spikes and gradual transitions while remaining robust to noise. Experiments on three subsets derived from physical full-scale crash tests show that MVCA-AD improves Precision, Recall, F1-score, and area under the ROC curve (AUC) over strong baselines and achieves stable performance under event-level grouped evaluation across heterogeneous head and B-pillar crash-test signals. The proposed approach supports crash-test data quality control by automatically identifying abnormal curves for downstream crashworthiness assessment workflows.

## 1. Introduction

Full-scale automotive crash tests underpin vehicle safety evaluation and crashworthiness assessment, and reliable conclusions depend on the integrity of sensor-recorded time series curves. Ensuring such integrity often requires time series anomaly detection, which plays a critical role across various engineering domains [1,2,3]. One representative application arises in automotive crash safety testing. During crash tests, a large number of sensors record physical quantities such as acceleration and force, generating complex time series known as “crash-test curves” [4]. Identifying anomalies within these curves is essential for distinguishing abnormal responses from instrumentation/data-acquisition issues that may compromise test interpretation, thereby supporting reliable crashworthiness assessment and vehicle safety system evaluation [5]. However, conducting full-scale crash tests is prohibitively expensive. Typically, only a limited number of tests can be performed per vehicle model, each requiring the destruction of a complete vehicle at high cost [6,7]. Meanwhile, labeled abnormal crash-test curves remain limited in absolute number, creating moderately imbalanced and small-sample labeled data [8,9]. These factors pose significant challenges for deep learning-based anomaly methods, which are known to be sensitive to data scarcity and distribution shift [10]; models must be trained on limited and imbalanced data while still generalizing to new vehicles and sensor configurations.

Conventional deep learning approaches have been widely applied to time series anomaly detection [11,12] and can be broadly categorized into the following types:

1. Reconstruction-based methods, such as AutoEncoders (AEs) and Variational AutoEncoders (VAEs), which detect anomalies via reconstruction errors [13,14,15,16];
2. Prediction-based methods, which use models like Long Short-Term Memory (LSTM) [17,18], Gated Recurrent Unit (GRU) [19], or Temporal Convolutional Network (TCN) [20] to predict future sequences and identify deviations based on prediction error;
3. Representation learning methods, including Deep Support Vector Data Description (Deep SVDD) [21], contrastive learning [22,23], and energy-based models [24], which learn discriminative features in an unsupervised or self-supervised manner.

Recently, Transformer-based architectures have also been introduced into time series anomaly detection [25]. Models such as Anomaly Transformer [26] and TranAD [27] leverage self-attention mechanisms to enhance the modeling of long-range dependencies and contextual shifts, improving the detection of sparse anomalies.

In related domains such as mechanical fault detection and structural health monitoring, previous studies have introduced engineered features like multi-scale convolution, and wavelet analysis to better capture shock, vibration, and trend behaviors [28,29,30]. These works suggest that physically interpretable time–frequency transformations can improve model robustness and help mitigate the scarcity of anomalous samples.

However, many existing time series anomaly detection methods are designed for multivariate settings or require rich contextual information, making them unsuitable for crash-test curves that often consist of single-channel measurements with extremely limited anomaly supervision [31,32]. In addition, crash-test curves collected from different vehicles and sensor locations exhibit substantial distribution shifts, and most models struggle to generalize across such conditions, limiting their applicability in real engineering workflows. Specifically, prior approaches suffer from (i) dependence on multi-sensor input, (ii) weak interpretability of learned features, and (iii) poor transferability across crash configurations.

This paper focuses explicitly on anomaly detection for single-channel crash-test curves. To address limitations related to anomaly scarcity, insufficient feature diversity, and poor generalization in existing methods, we propose MVCA-AD (Multi-View Context Augmentation for Anomaly Detection). The key idea of MVCA-AD is to construct physically interpretable multi-view representations for each input signal, explicitly modeling anomaly patterns across different time scales. For instance, given a raw acceleration signal as the anchor view, we derive its first-order derivative (emphasizing sharp changes), integral (capturing low-frequency response), spectrum and time–frequency maps (revealing vibration modes), and higher-order derivatives (enhancing impulsive transients). These deterministic views carry well-defined physical semantics and amplify anomalies at different scales, offering a rich and complementary feature basis for detection.

In terms of architecture, MVCA-AD assigns an independent encoder to each view and introduces a Feature-wise Linear Modulation (FiLM) mechanism to perform scale and distribution calibration using the anchor view as the conditioning context. This aligns the statistical properties across different views. Next, a multi-head cross-view attention module is employed to dynamically fuse features across views, adaptively selecting the most informative perspective at each time step—e.g., derivative views for shock segments and integrals for slow dynamics. The fused temporal embeddings are then fed into a feedforward classifier for sequence-level anomaly prediction. The entire pipeline is jointly optimized rather than trained as isolated modules, ensuring cooperative feature learning.

MVCA-AD offers the following key advantages: (i) It eliminates the need for multichannel setups, enabling anomaly detection from a single sensor channel, which simplifies use in single-sensor settings. (ii) By incorporating prior knowledge such as channel names, the model learns patterns that remain stable across heterogeneous vehicle-test events and sensor channel types under group-preserving evaluation. (iii) It robustly handles data imbalance by leveraging multi-view augmentation to explicitly expose rare anomaly patterns, thereby demonstrating strong performance on physical full-scale crash-test data.

In summary, the contributions are as follows:

- We propose MVCA-AD, a multi-view anomaly detection framework tailored for single-channel crash-test curves, combining physical signal transformations with deep representation learning;
- We design a cross-view dynamic fusion strategy and a trend-aware modulation module to enhance sensitivity to multi-scale dynamics and transient anomalies.
- We demonstrate stable performance under event-level grouped evaluation, with MVCA-AD maintaining high detection stability across heterogeneous head and B-pillar sensor subsets within the scope of the evaluated dataset, addressing the practical applicability gap in engineering workflows.

Subsequent sections will elaborate on the MVCA-AD architecture, experimental setup, and analysis of results.

## 2. Technological Background

This study addresses the challenge of enhancing single time series representations by constructing deterministic multi-view expansions and modeling cross-view feature interactions. In the proposed framework, each sensor curve is transformed into a set of complementary views in both time and frequency domains, capturing derivative, integral, and oscillatory characteristics of the same signal. The objective is to detect anomalous responses in automotive crash-test curves through a backbone based on dilated residual convolutions, augmented with Feature-wise Linear Modulation (FiLM) and attention-based fusion to enable adaptive feature recalibration across views. Accordingly, this section outlines the key theoretical foundations relevant to the proposed framework, including Multi-View Context Augmentation, the FiLM mechanism, attention mechanisms, and the dilated residual convolution network.

### 2.1. Multi-View Context Augmentation

Single-channel time series signals often exhibit heterogeneous temporal dynamics that coexist across multiple scales, including sharp high-frequency transients, mid-range structural oscillations, and slow-varying global trends [33]. When such diverse patterns are compressed into a single raw curve, a learning model must implicitly infer all relevant temporal structures from one representation. This often limits feature separability and weakens the model’s ability to capture subtle deviations, particularly when training samples are limited or contaminated by noise [34].

To expose multi-scale characteristics explicitly, Multi-View Context Augmentation constructs a set of complementary representations—referred to as “views”—through deterministic transformations. Formally, given an input time series signal x(t), a set of n complementary views is constructed through deterministic transformations:

(1) $$V\left(x\right)={\left\{v_{k}\left(t\right)\right\}}_{k=1}^{n},\;\;v_{k}\left(t\right)=T_{k}\left(x\left(t\right)\right),\;\;k=1,\ldots ,n$$

where $T_{k}$(·) denotes a deterministic transformation operator applied to the input signal x(t), and V(x) denotes the resulting set of all n derived views. Commonly adopted operators include time-domain transforms (e.g., derivatives, cumulative integrals, smoothing, trend extraction) and time–frequency transforms (e.g., short-time Fourier analysis, wavelet-based decompositions, or envelope detection) [35,36]. In mechanical and structural systems, such multi-resolution representations are widely employed to reveal impact shocks, vibration modes, damping behaviors, and energy-dissipation characteristics that are not readily observable from the raw signal alone. Prior studies in condition monitoring, fault diagnosis, and time series representation learning have demonstrated that combining raw signals with their derivative, integral, or spectral forms improves robustness and discriminative ability [29].

Compared with stochastic data augmentation, this multi-view construction is fully deterministic and physically interpretable: each view corresponds to a specific temporal property of the underlying physical process. This property is advantageous for anomaly detection, where abnormal events often manifest as subtle shifts in timing, amplitude modulation, irregular bursts, or oscillatory content—patterns that may emerge more clearly in certain transformed domains.

In the context of automotive crash-test signals, the need for multi-view augmentation is particularly pronounced. Crash-response curves are non-periodic, strongly impulsive, and highly non-stationary: shock-induced spikes and low-frequency structural oscillations coexist within milliseconds, and channel-wise baseline/trend differences are substantial. Moreover, abnormal crash events exhibit common temporal signatures across channels, but are embedded within otherwise sparse and low-density information. A deterministic multi-view expansion allows these heterogeneous temporal patterns to be decomposed and exposed explicitly, enabling the downstream model to learn channel-agnostic, anomaly-sensitive representations more effectively.

In this study, each crash-test signal is expanded into a compact set of deterministic views that emphasize different temporal scales and dynamical behaviors. The specific instantiation of these views and their integration within the MVCA-AD framework are described in detail in Section 3.

### 2.2. Feature-Wise Linear Modulation (FiLM)

Feature-wise Linear Modulation (FiLM) [37] is a general conditioning mechanism that enables neural networks to adapt intermediate feature activations according to external or contextual information. FiLM introduces a simple yet effective affine transformation applied to each feature channel, enabling the model to dynamically reparameterize its internal representation.

Formally, given an intermediate feature tensor $F\in R^{C\times L}$ and a conditioning vector z, FiLM applies channel-wise scaling and shifting operations as

(2) $$FiLM\left(F|z\right)=\gamma \left(z\right)⊙F+\beta \left(z\right),$$

where $\gamma \left(z\right)$ and $\beta \left(z\right)$ denote the modulation coefficients generated by a separate conditioning network, and ⊙ represents element-wise multiplication. The coefficients are learned jointly with the backbone parameters through standard gradient-based optimization, allowing flexible adaptation of the network’s internal statistics to different input contexts.

The key advantage of FiLM is its ability to perform contextual recalibration without altering the overall network topology. It introduces minimal computational overhead while enabling task-specific or context-specific adaptation of shared representations. In time series modeling, FiLM allows different temporal patterns—such as abrupt impulses or slowly varying trends—to be processed under distinct scaling and shifting conditions, thus enhancing representational flexibility and improving generalization across heterogeneous signal behaviors.

In the MVCA-AD framework, FiLM serves as a lightweight conditioning module that modulates feature responses from different deterministic views, aligning their statistical scales and facilitating subsequent cross-view fusion. The implementation details and integration strategy are discussed in Section 3.

### 2.3. Attention Mechanism

Attention mechanisms have become a cornerstone in deep representation learning for capturing context-dependent relationships within sequential or spatial data [38]. Rather than processing all elements uniformly, attention assigns adaptive weights that quantify the relative importance of each input feature when forming contextual representations. This selective focus enables neural networks to model long-range dependencies and complex interactions that purely convolutional or recurrent structures often struggle to capture.

The canonical scaled dot-product attention computes a weighted combination of value vectors V based on the similarity between a query Q and a set of keys K:

(3) $$Attention\left(Q,K,V\right)=softmax\left(\frac{QK^{T}}{\sqrt{d_{k}}}\right)V,$$

where $d_{k}$ denotes the dimension of the key vector for normalization. The softmax operation produces a probability distribution emphasizing the most relevant elements for each query while suppressing less informative ones.

To capture diverse correlation patterns, multi-head attention extends this formulation by projecting the input into multiple subspaces, each learning an independent attention map. This allows the model to simultaneously attend to features at different temporal scales or semantic levels. In contrast, cross-attention leverages distinct query and key-value sources, enabling information exchange between different feature domains—such as between raw and transformed signal views in a multi-view framework.

For time series applications, attention provides a flexible mechanism for identifying salient events and aligning asynchronous temporal patterns [26,27]. By assigning higher weights to critical signal segments (e.g., impact impulses or abrupt amplitude changes) while maintaining global temporal awareness, attention facilitates robust and interpretable feature integration.

Within the MVCA-AD framework, a multi-head cross-attention module is adopted to realize fine-grained information fusion among deterministic views. Each attention head captures complementary dependencies across temporal representations, enhancing the model’s capacity to correlate physical dynamics observed in different transformation domains. The detailed formulation and its role in the proposed architecture are presented in Section 3.

### 2.4. Dilated Residual Convolution Network (DRN)

Convolutional architectures are widely used in time series modeling for their ability to efficiently capture local temporal patterns. A representative example is the Temporal Convolutional Network (TCN) [39], which employs dilated causal convolutions to enlarge the receptive field while restricting each output to depend solely on past observations. Such a causal structure is essential in forecasting problems, where future information must not influence model predictions.

However, sequence-level anomaly detection—as in this work—differs fundamentally from forecasting. The full time window (e.g., 0–200 ms after impact) is available before inference, and the objective is not to predict future values but rather to extract discriminative features from the entire segment. Enforcing causality in this setting prevents the model from leveraging informative structures that occur after salient events such as the impact onset, rebound oscillations, or secondary structural responses. To enable full-context reasoning, we replace causal TCN layers with a Dilated Residual Network (DRN) [40], which retains the multi-scale advantages of dilation while discarding causal padding.

The core operation of DRN is the dilated 1D convolution. For a kernel ω of size K and dilation factor d, the dilated convolution is defined as

(4) $$\left(x\times_{d}\omega \right)\left(t\right)=\sum_{k=0}^{K-1}\omega \left(k\right)x\left(t-dk\right),$$

Equation (4) is shown in causal form for notational clarity; the actual implementation uses symmetric (non-causal) padding so that each output draws on both past and future samples within the receptive field. This dilation increases the effective receptive field by a factor of d without introducing additional parameters. By stacking multiple layers whose dilation rates grow exponentially (e.g., d∈{1,2,4,…}), the model becomes sensitive to patterns that unfold over fine-to-coarse temporal scales.

For an L-layer stack with kernel sizes $K_{l}$ and dilations $d_{l}$, the total receptive field is

(5) $$R=1+\sum_{l=1}^{L}{(K}_{l}-1)d_{l},$$

To stabilize training in deep dilation stacks, DRN employs residual connections. For the l-th block, the formulation is as follows:

(6) $$y^{\left(l\right)}=x^{\left(l\right)}+F^{\left(l\right)}\left(x^{\left(l\right)}\right),$$

where $F^{\left(l\right)}(\cdot )$ consists of a dilated convolution followed by normalization and nonlinear activation. Residual pathways allow the network to simultaneously learn low-frequency structural trends and high-frequency impact transients while mitigating gradient degradation, which is essential for datasets of limited size such as physical full-scale crash tests.

In summary, DRN provides a computationally efficient, fully convolutional backbone that preserves multi-scale temporal sensitivity while enabling bidirectional context aggregation. These properties align naturally with the characteristics of crash-test signals—strongly impulsive, non-periodic, and highly non-stationary—and make DRN a suitable choice for sequence-level anomaly classification in the MVCA-AD framework.

## 3. Proposed MVCA-AD

### 3.1. Framework Overview

MVCA-AD is an end-to-end multi-view representation learning framework tailored for sequence-level anomaly detection in crash-test acceleration curves. The model transforms each raw input signal through four stages:

1. Deterministic multi-view expansion, which generates a set of physically interpretable temporal and spectral views;
2. View-wise temporal encoding via dilated residual convolutions;
3. Trend-conditioned FiLM alignment for adaptive feature recalibration across views;
4. Multi-head cross-view attention fusion to integrate complementary temporal cues into a unified representation.

**Figure 1:**
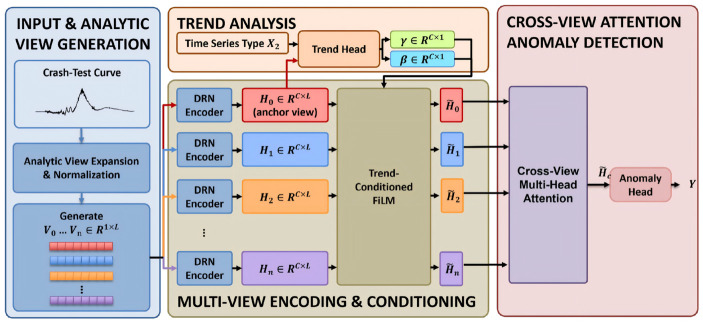
The overall framework of MVCA-AD.

All modules are jointly trained under a unified objective to ensure cooperative learning of transformation-specific features, modulation dynamics, and cross-view interactions. The resulting fused feature vector is passed to a lightweight multilayer perceptron (MLP) for final anomaly classification. The overall architecture is illustrated in Figure 1.

### 3.2. Deterministic Multi-View Expansion

MVCA-AD captures multi-scale temporal dynamics by introducing a library of deterministic and physically interpretable transforms (Table 1) that convert a raw single-channel input signal $x\in R^{1\times L}$ into a set of complementary views ${\{V}_{k}\}$ across time and frequency domains. These transformations fall into three categories:

Throughout this section, we use $R^{C\times L}$ to denote a real-valued tensor with C channels and L temporal samples; the multi-view stack across V views has shape $R^{V\times C\times L}$. When C=1 (e.g., the input signal x or any deterministic view $V_{k}$), the tensor reduces to $R^{1\times L}$. Encoded feature maps $F_{k}$ from the view-specific encoders take the shape $R^{C\times L}$ with C>1; their stack across views, used by the cross-view attention module, takes the shape $R^{V\times C\times L}$.

Temporal derivative views (ID = −1, −2): The first derivative highlights abrupt changes in velocity or impact, helping detect slope transitions at the onset of collision. The second derivative captures curvature, which is useful for identifying nonlinear changes such as peaks or inflection points. Together, they characterize rapid dynamic responses in the signal.

Integral views (ID = 1, 2): Integration captures the accumulated trend of the signal, analogous to physical notions like momentum or energy buildup. The first integral emphasizes overall speed patterns, while the second captures displacement or long-term trends, useful for modeling post-collision stabilization.

Frequency-domain filtering views:

The Fourier filter bank (ID = 10) decomposes the signal into multiple frequency bands and extracts magnitude envelopes, emphasizing energy distributions across spectral components.

Specifically, the Fourier filter bank is implemented as a parallel cosine/sine convolution followed by magnitude pooling, log-compression, and a learnable 1 × 1 mixing layer. Given an input signal x(t) sampled at frequency $f_{s}$, the bank uses $N_{F}=12$ frequency bands whose center frequencies $\left\{f_{b},b=1,\ldots ,N_{F}\right\}$ are logarithmically spaced between $f_{min}=1\;Hz$ and $f_{max}=min(500\;Hz,0.9f_{s}/2)$. For each band b, a Hann-windowed cosine–sine kernel pair of length $K_{F}\approx 0.020f_{s}$ samples (a 20 ms analysis window, rounded to the nearest odd integer) is constructed and pair-normalized to unit energy. The per-band magnitude is computed as

(7) $$m_{b}\left(t\right)=\sqrt{{\left(c_{b}\times x\right)}^{2}\left(t\right)+{\left(s_{b}\times x\right)}^{2}\left(t\right)},$$

where × denotes 1D convolution along the temporal axis; $m_{b}$ is then smoothed by a 5 ms moving-average kernel, log-compressed via $log(1+m_{b})$, and aggregated across the $N_{F}=12$ bands by a learnable 1 × 1 convolution into a single-channel output of the same length L as x.

The Morlet continuous wavelet transform (CWT, ID = 11) offers time–frequency localization, ideal for capturing short-term transients or multi-scale oscillations around the impact point.

The Morlet continuous wavelet transform is implemented analogously with $N_{W}=16$ scales whose center frequencies $\left\{f_{j},j=1,\ldots ,N_{W}\right\}$ are logarithmically spaced over the same $\left[f_{min},f_{max}\right]$ interval as the Fourier bank. Each scale j uses a complex Morlet wavelet

(8) $$\psi_{j}\left(t\right)=g_{j}\left(t\right)\cdot e^{i\;\cdot \;2\pi f_{j}t},$$

with Gaussian envelope

(9) $$g_{j}\left(t\right)=exp\left(-t^{2}/\left(2{\sigma_{j}}^{2}\right)\right),$$

whose bandwidth

(10) $$\sigma_{j}=\frac{N_{c}}{2\pi f_{j}},$$

is controlled by $N_{c}=6$ wavelet cycles, providing approximately constant-Q time–frequency localization. The cosine and sine components of $\psi_{j}$ are pair-normalized to unit energy, convolved with x, magnitude-pooled, log-compressed via log(1+⋅), and aggregated across the $N_{W}=16$ scales by a learnable 1 × 1 convolution into a single-channel output of the same length L as x, mirroring the Fourier bank pipeline above.

The Butterworth low-pass filter (ID = 21) suppresses high-frequency noise and preserves slow trends, aiding in the interpretation of smooth structural responses.

Additionally, the anchor view (ID = 0) retains the original acceleration curve as a temporal reference. All transformed views are deterministic and yield output sequences of equal length as the input. Thus, each view produces a tensor $V_{k}\in R^{1\times L}$, enabling straightforward stacking into a unified representation $R^{V\times 1\times L}$ without alignment or resampling, making them readily processable by view-specific temporal encoders that share a common architectural design (see Section 3.3).

**Figure 2:**
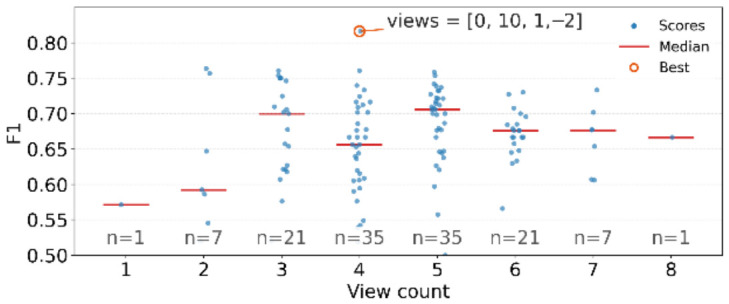
F1-scores of MVCA-AD under different multi-view combinations.

As shown in Figure 2, we systematically evaluated how view composition affects performance. With the anchor view (ID = 0) fixed in all configurations, we enumerated all 128 subsets of the remaining seven views from the transform library. Each was tested on the validation set, and F1-scores are visualized as blue points, with red lines denoting medians per-view count.

The optimal performance was achieved with four views: [0, 10, 1, −2]—the raw signal, Fourier magnitude, first integral, and second derivative—demonstrating that combining complementary physical transformations is crucial for discriminative anomaly detection.

### 3.3. View-Specific Dilated Residual Encoders

**Figure 3:**
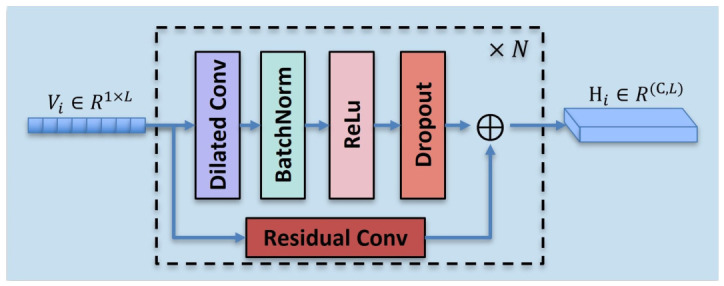
View-specific dilated residual encoder architecture for temporal modeling.

To extract temporal patterns from each transformed view $V_{i}\in R^{1\times L}$, MVCA-AD employs a view-specific dilated residual encoder (illustrated in Figure 3), where each view is assigned a dedicated network with distinct parameters despite sharing the same architectural design. The encoder consists of eight stacked 1D residual blocks, with dilation rates following the sequence [1, 2, 4, 8, 16, 32, 64, 128]. This exponentially expanding dilation enables a large receptive field of 511 timesteps, covering long-range dependencies while preserving the input resolution. Within each residual block, a 1D dilated convolution (kernel size = 3, padding = dilation) is followed by Batch Normalization, ReLU activation, and dropout (rate = 0.1). Channel dimensions increase progressively from 32 to 128, with a 1 × 1 convolution applied in the residual path when channel projection is required. The final output $H_{i}\in R^{C\times L}$ is a high-dimensional sequence that captures rich temporal responses specific to each view. By allocating separate encoders, MVCA-AD accommodates diverse statistical characteristics across transformations, enhancing both representational flexibility and discriminative power.

### 3.4. Trend Head and FiLM-Based View Alignment

**Figure 4:**
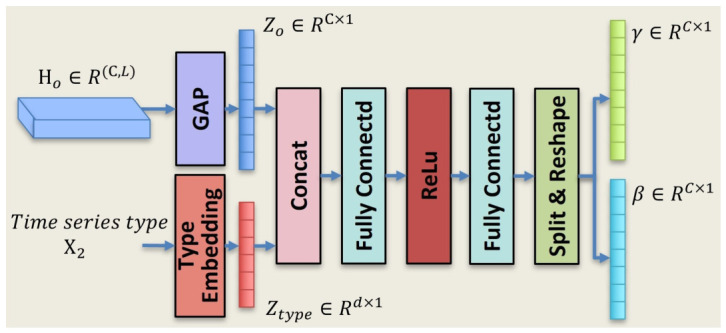
FiLM-based view feature modulation.

Crash-test curves often exhibit varying long-term trends depending on the sensor type. To address this variation and enhance the detection of shared anomaly patterns, a FiLM (Feature-wise Linear Modulation) mechanism is employed, as illustrated in Figure 4. This modulation dynamically applies learned channel-wise scaling and bias to emphasize anomaly-relevant signals while suppressing type-specific drift.

The process begins with the anchor view (view ID = 0), which retains the raw acceleration curve. After temporal encoding, the anchor feature map $H_{0}\in R^{C\times L}$ undergoes global average pooling along the temporal dimension, yielding a channel-level context vector $z_{0}\in R^{C\times 1}$ that summarizes the sequence trend.

Simultaneously, the model incorporates a known curve-type prior that describes the physical origin of the input signal (e.g., dummy head acceleration in the X direction). This type name is embedded into a dense vector $z_{type}\in R^{d\times 1}$ via a learnable embedding layer and concatenated with the anchor summary $z_{0}$ to form the modulation input.

The concatenated vector $z_{in}=concat\left(z_{0},z_{type}\right)\in R^{\left(C+d\right)\times 1}$ is then passed through two fully connected layers to produce channel-wise FiLM parameters: a scaling factor $\gamma \in R^{C\times 1}$ and a bias term $\beta \in R^{C\times 1}$. These parameters are broadcast and applied to each view-specific feature map $H_{v}$ as

(11) $${\tilde{H}}_{v}=H_{v}⊙\gamma +\beta ,$$

This conditioning mechanism allows MVCA-AD to align feature distributions across views while compensating for inter-type variations. The recalibrated feature maps ${\tilde{H}}_{v}$ are then forwarded to the cross-view attention fusion module for joint modeling.

### 3.5. Cross-View Fusion and Anomaly Classification

**Figure 5:**
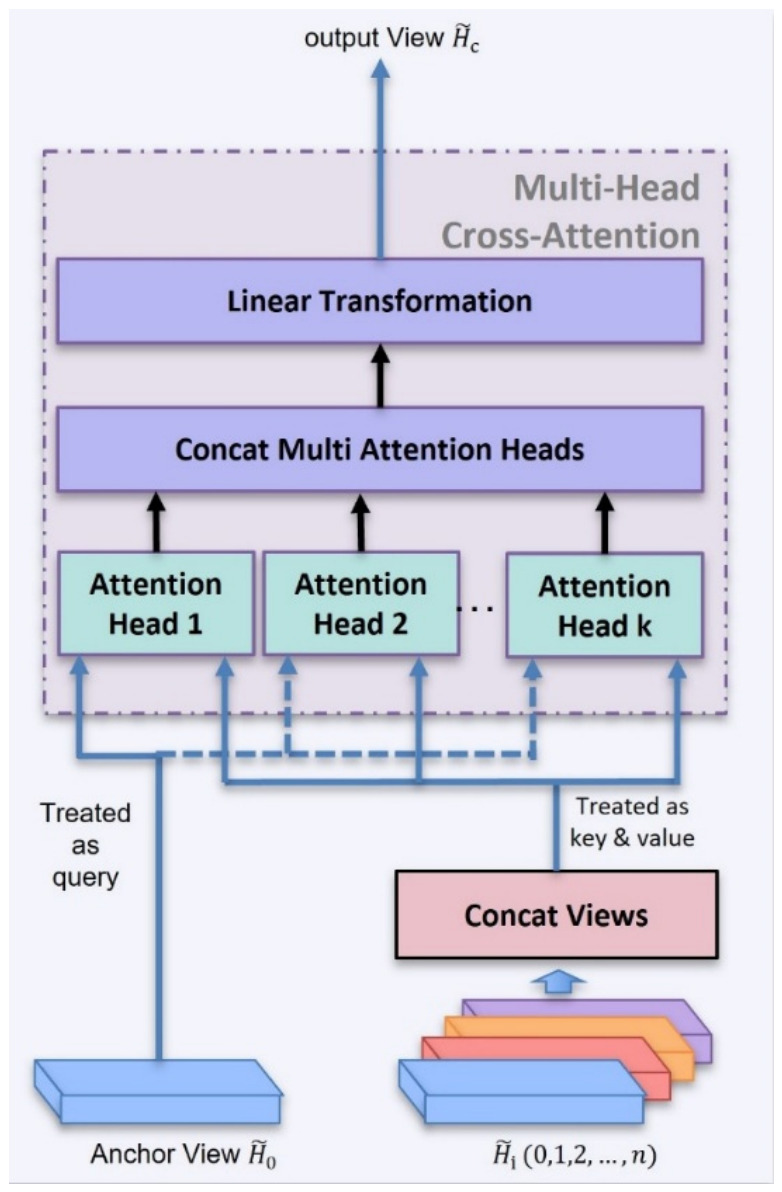
Multi-head cross-view attention fusion module.

The final integration of multi-view information relies on a per-time-step multi-head cross-view attention fusion module (as illustrated in Figure 5), which fuses dynamic features across different views. The anchor view serves as the query source: its encoded feature map ${\tilde{H}}_{0}\in R^{C\times L}$ is first passed through a 1 × 1 convolution and transposed along the temporal dimension to produce a query sequence $Q\in R^{L\times C}$, representing time-specific context queries.

Simultaneously, the encoded features from all views ${\left\{{\tilde{H}}_{v}\right\}}_{v=0}^{V}$ are stacked along the view dimension to form a key-value tensor $KV\in R^{L\times V\times C}$, and a learnable view embedding is added as bias. For each time step, an eight-head attention mechanism is applied between the anchor query and the stacked key-value pairs across views, resulting in an aggregated representation $\hat{H}\in R^{L\times C}$.

This output is further refined via residual connection, layer normalization, and a two-layer feedforward network (FFN) with Gaussian Error Linear Unit (GELU) activation and dropout. The refined output is finally projected back to the original channel format, producing the fused representation ${\tilde{H}}_{c}\in R^{C\times L}$, which is then forwarded to the pooling module for final anomaly classification.

After multi-view feature fusion, MVCA-AD employs a lightweight anomaly head to perform binary classification over the entire sequence. The fused representation ${\tilde{H}}_{c}$ is first aggregated by a hybrid temporal pooler combining adaptive average pooling and attention pooling, producing a fixed-dimensional feature vector. This vector is then passed through a shallow two-layer feedforward network to predict the anomaly confidence score of the input sequence.

The module outputs a scalar logit that indicates the likelihood of the sequence being anomalous, which is subsequently used in loss computation. The anomaly head does not incorporate auxiliary branches or explicit temporal attention mechanisms; all temporal dynamics are modeled implicitly by the preceding encoder and fusion modules. This design choice simplifies the learning objective and contributes to more stable training convergence.

## 4. Case Studies and Results

### 4.1. Experimental Setup

#### 4.1.1. General Settings

All experiments are implemented in PyTorch 2.5.1+cu121, using Python 3.11, and executed on a workstation equipped with an Intel Core i9-14900K CPU (Intel Corporation, Santa Clara, CA, USA), 192 GB of RAM, and an NVIDIA RTX 4090 GPU (NVIDIA Corporation, Santa Clara, CA, USA).

The models are optimized using the Adam optimizer with a learning rate of 3 × 10^−4^, trained using the binary cross-entropy loss with logits. A batch size of 64 and a cosine learning rate schedule are adopted. Given the relatively small dataset sizes (see Section 4.1.2), we set the maximum number of training epochs to 30 to reduce the risk of overfitting. Early stopping with a patience of five epochs is applied.

To address the class imbalance inherent in the anomaly detection setting, we incorporate a positive class weighting term computed solely from the training split. Model selection follows the validation F1@0.5 criterion, which provides a balanced operating point between Precision and Recall under a fixed decision threshold.

To prevent leakage between the held-out evaluation partition and any model-selection decision, all reported metrics follow strict nested partitioning. For each random seed, the crash-test events were first split at the event level into a training partition and a held-out evaluation partition (80/20). The held-out evaluation partition was used exclusively for the final reported metrics in Table 2 and Table 3 and was never used for view selection, early stopping, threshold selection, or any other model-selection decision. Within the training partition, a further event-level split provided an internal validation subset, which was used solely for early stopping and model selection according to F1@0.5. The view composition search reported in Figure 2 was performed exclusively on internal validation splits derived from the training partition; the final selected view set [0, 10, 1, −2] was fixed before evaluation on the held-out partition.

#### 4.1.2. Data Preparation

The automotive crash-test time series dataset used in this study was obtained from a vehicle safety research facility located in Jilin Province, China. The data used in this study come from 105 routine physical full-scale crash tests conducted by the R&D center. Due to confidentiality constraints, the data provider does not disclose detailed information regarding the exact test conditions, vehicle platforms, and occupant dummy specifications. All recordings nevertheless follow the standardized full-scale crash-test protocols routinely used in vehicle passive-safety evaluation (consistent with China New Car Assessment Program (C-NCAP)-class evaluation procedures), with sensor instrumentation and signal conditioning conforming to the Society of Automotive Engineers (SAE) J211-1 specification, including its specified Channel Frequency Class filtering.

The dataset comprises crash-test records from a range of passenger vehicles, encompassing various body types and design generations. Vehicle platform identifiers were anonymized by the data provider; the exact number of distinct platforms is therefore not available for disclosure, and vehicle identity was not used as an input feature or stratification variable. Each test vehicle was equipped with onboard sensors installed at key structural and occupant-relevant positions to capture dynamic responses throughout the crash event. Each crash sample is recorded as a set of synchronized multichannel time series. Among all available measurement channels, two representative sensing locations are selected for analysis: the driver head region (head) and the B-pillar structure (B-pillar), which are both widely recognized as critical indicators in crash-response evaluation for assessing occupant safety and structural integrity.

The total number of time series signals used in this study is 662, including 252 channels from the dummy head and 410 channels from the vehicle B-pillar. The sensors were sampled at 20 kHz, resulting in 20,000 data points covering a 1 s window from −500 ms to +500 ms relative to the impact trigger. For analysis, a 0–200 ms segment following the crash onset is extracted (4000 samples). This time window captures the main deformation and occupant response phase of the frontal crash event, while excluding post-impact oscillations irrelevant to anomaly detection.

All crash tests in this dataset were conducted under the full-width rigid barrier (FRB) frontal impact configuration, following the China New Car Assessment Program (C-NCAP) protocol. The nominal impact speeds are 50 kph and 55 kph. Each test vehicle is instrumented with a Hybrid III 50th-percentile male anthropomorphic test device (ATD). The available instrumentation included up to 21 acceleration channels capturing triaxial (X/Y/Z) acceleration at five body regions: driver head (position 11), passenger head (position 13), rear-seat occupant head, near-side B-pillar lower and middle sections (position 14), and far-side B-pillar lower and middle sections (position 16), all acquired at 20 kHz and filtered in accordance with SAE J211-1.

Each signal is annotated with a single binary class label by passive-safety domain experts from the data provider, who classified each curve as either normal (a standard-compliant crash response) or anomalous (exhibiting signal-level deviations or performance violations). The annotation is whole-curve, with no segment-level intervals and no further sub-categorization of anomaly type. This binary labeling protocol reflects the operational practice in crash-test laboratories, where the primary objective is rapid screening: engineers flag any curve whose morphology deviates from the expected crash response—whether the deviation originates from sensor malfunction, data-acquisition errors, or genuinely atypical structural responses—so that flagged signals can be reviewed and, if necessary, the test repeated. Finer-grained anomaly categorization (e.g., distinguishing acquisition faults from performance anomalies) would require additional metadata such as sensor diagnostic logs, which fall outside the scope of the present dataset. Across the 662 signals, the resulting class distribution is 503 normal and 159 anomalous (anomaly rate 24.0%). By subset, the head subset contains 252 signals (209 normal/43 anomalous, 17.1%), the B-pillar subset contains 410 signals (294 normal/116 anomalous, 28.3%), and the Combined subset aggregates both. The labeling decisions reflect the experts’ professional assessment based on each curve’s morphological features (impact spike timing, baseline drift, and signal artifacts). Vehicle identity and crash-test ID were not used as explicit input features for model training.

**Figure 6:**
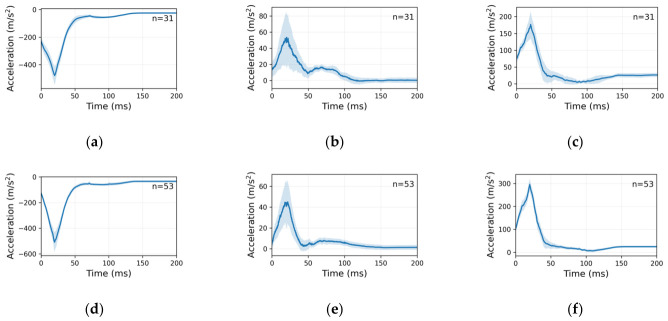
Time-aligned head acceleration responses (mean ± 95% CI) for driver and passenger dummies: (**a**–**c**) driver head X/Y/Z; (**d**–**f**) passenger head X/Y/Z.

**Figure 7:**
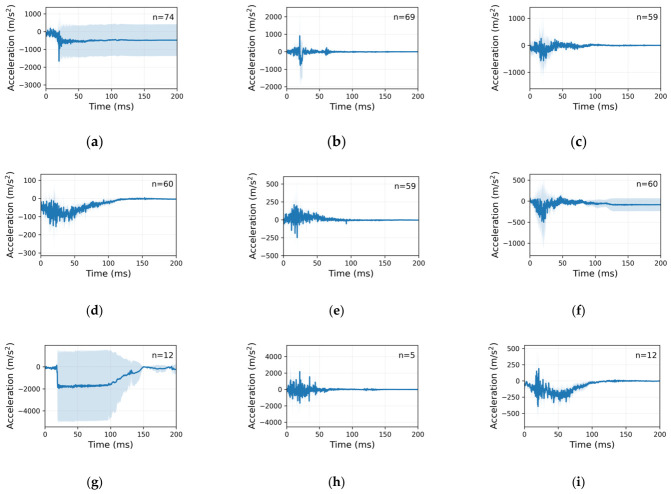
Time-aligned B-pillar acceleration responses (mean ± 95% CI): (**a**–**c**) left-bottom X/Y/Z; (**d**–**f**) right-bottom X/Y/Z; (**g**,**h**) left-middle X/Y; (**i**) right-middle X.

Specifically, the dataset comprises time series signals from the driver’s head, passenger’s head, left B-pillar mid-section, left B-pillar lower section, right B-pillar mid-section, and right B-pillar lower section. For clarity of visualization, each subplot uses an independent y-axis range that reflects the physical magnitude of the corresponding sensor channel. The averaged acceleration responses of these sensing locations (mean ± 95% confidence interval (CI)) are presented in Figure 6 and Figure 7, corresponding to the head and B-pillar sensors, respectively.

Before model training, each sequence is independently normalized using its own mean and standard deviation (no statistics from other samples or from the held-out evaluation partition are used), and length-aligned. Anomalous samples correspond to test runs exhibiting signal-level deviations or performance violations identified by domain experts, whereas normal samples represent standard-compliant crash responses. To evaluate cross-event evaluation performance, the dataset is partitioned at the crash-test event level rather than at the signal level: each crash-test event is assigned in its entirety to exactly one of the training or evaluation partitions, so signals from the same crash-test event never appear simultaneously across the two sets. Five independent random seeds (42, 123, 2024, 3407, 7) are used to produce 80/20 train/evaluation partitions, and all reported metrics are independently averaged over the five splits. The master split is performed on the Combined subset (containing all 105 distinct crash-test events) and projected onto the head and B-pillar subsets to maintain methodological consistency across all three experimental subsets. All reported metrics (Precision, Recall, F1@0.5, ROC-AUC) are independently averaged over the five splits. Note that, under group-based splitting, the exact sample-level partition ratio cannot be perfectly preserved due to the variable number of channels per crash-test event (3 to 14) and the non-uniform anomaly distribution across events; the resulting evaluation set sizes vary across splits (Combined: 120~130 signals, B-pillar: 61~82, head: 42~69).

#### 4.1.3. Evaluation Metrics

Model performance is quantified using four widely adopted metrics in anomaly detection studies—Precision, Recall, F1-score (computed at a fixed threshold of 0.5), and the area under the Receiver Operating Characteristic curve (ROC-AUC). Predictions are categorized into four outcomes: true positives (TPs), false positives (FPs), true negatives (TNs), and false negatives (FNs), with the anomalous class treated as the positive class in this binary classification setting.

**Precision.** Proportion of samples predicted as anomalous that are actually anomalous:

(12) $$Precision=\frac{TP}{TP+FP},$$

**Recall.** Proportion of anomalous samples that are correctly identified:

(13) $$Recall=\frac{TP}{TP+FN},$$

**F1-score.** The F1-score is defined as the harmonic mean of Precision and Recall:

(14) $$F1=\frac{2Precision\cdot Recall}{Precision+Recall}=\frac{2TP}{2TP+FP+FN},$$

**ROC-AUC.** A threshold-independent ranking metric obtained by sweeping the decision threshold to form the Receiver Operating Characteristic (ROC) curve and integrating the area under its trajectory of the true positive rate versus the false positive rate. 

To ensure fairness and consistency, all models are evaluated using the same metric settings on the held-out evaluation sets across the five group-preserving splits. Each seed controls both the event-level grouped split and model initialization; the reported standard deviations therefore quantify total variability across the five independent runs. Higher values of Precision, Recall, F1-score, and ROC-AUC indicate better performance.

### 4.2. Baseline Comparison

To evaluate MVCA-AD, we compare it with nine baseline methods grouped into three methodological categories: classical machine learning models, convolution-based models, and recurrent models.

**Classical machine learning model.** This category includes non-deep approaches based on feature transformations. We adopt RandOm Convolutional KErnel Transform (ROCKET) with logistic regression [41], where large sets of random convolutional kernels produce proportion-of-positive-values (PPV) features that are subsequently classified using a linear model.

**Convolution-based models.** These methods extract temporal features through hierarchical convolutional filters with different receptive fields. The baselines in this category include Convolutional Neural Network (CNN), Fully Convolutional Network (FCN), ResNet [42,43], DRN [40], InceptionTime [44], and TCN [39], all of which perform direct 1D convolutional processing on the raw time series signals.

**Recurrent models.** These models capture temporal dependencies through gated recurrent structures. We employ LSTM [45] and GRU [46] as representative recurrent baselines, both trained to model sequential patterns in the sensor signals.

All baselines are implemented and trained under the same data splits and metric settings as MVCA-AD to ensure comparability. The quantitative results on the three datasets are summarized in Table 2. Several clear patterns emerge from the comparison:

1. Baseline methods exhibit strong dataset-dependence, with large performance fluctuations across datasets. Among baselines, InceptionTime remains the strongest, achieving F1 of 59.89% (B-pillar), 52.13% (head), and 39.33% (Combined), averaged over five random splits. Despite leading among baseline methods, its scores vary substantially across the three subsets, suggesting limited cross-dataset stability.
2. MVCA-AD consistently achieves the highest Recall on all datasets—62.23% (head), 83.90% (B-pillar), and 84.41% (Combined)—substantially reducing false negatives compared with the best baseline in each dataset. Since missed anomalies (FN) correspond to expert-flagged abnormal crash-test curves that would escape automatic screening, Recall preservation across five event-level grouped splits is operationally important.
3. The strongest gains occur on the “Combined” dataset. Since this dataset mixes heterogeneous signals from different sensors and vehicles, it serves as a more challenging within-dataset evaluation of the model’s ability to handle channel-type heterogeneity. In this challenging scenario, MVCA-AD achieves the best results across all four metrics—Precision (59.57%), Recall (84.41%), F1 (69.84%), AUC (84.46%)—maintaining a substantial lead over the strongest baseline (InceptionTime, F1 39.33%).

Across the four metrics, these results show that MVCA-AD not only provides the most stable performance across all datasets but also delivers the largest improvements precisely where cross-view heterogeneity is highest, supporting its robustness to event-level and channel-type heterogeneity within the evaluated dataset.

### 4.3. Ablation Study

To quantify the contribution of each component in MVCA-AD, we conduct a systematic ablation study by selectively enabling or disabling three key modules: (i) Multi-View Context Augmentation, (ii) FiLM-based affine modulation, and (iii) cross-view attention fusion. These three modules were selected as the ablation targets because they correspond to the three core architectural additions that distinguish MVCA-AD from a plain DRN backbone (introduced in Section 3.2, Section 3.4 and Section 3.5, respectively), and each plays a functionally distinct role: MVCA expands the feature space by augmenting the input with deterministic physical views, FiLM aligns the statistical scales of these views through type-aware affine modulation, and cross-view attention enables fine-grained information exchange across views. The three components are therefore minimally overlapping, allowing the contribution of each architectural innovation to be isolated and measured independently. All variants share the same DRN backbone to ensure comparability. The full results are presented in Table 3.

1. Effect of Multi-View Context Augmentation (MVCA)

Introducing MVCA alone leads to clear and consistent improvements across all three datasets, raising mean F1 from 42.38% to 62.00% on B-pillar, from 24.44% to 39.55% on head, and from 26.38% to 46.28% on Combined. The performance lift is most pronounced on the Combined dataset—where multi-view heterogeneity is highest—showing that MVCA effectively enriches single-curve representations by mining intrinsic information across views.

2. Effect of FiLM

FiLM alone also improves upon the DRN baseline on all three datasets—F1 rises from 42.38% to 45.04% on B-pillar, from 24.44% to 29.41% on head, and from 26.38% to 35.43% on Combined—demonstrating that class-aware and anchor-conditioned affine modulation provides additional representational flexibility even without auxiliary views. When FiLM is applied on top of MVCA, all three datasets exhibit further gains (F1 on Combined rises from 46.28% to 62.06%), reflecting a consistent pattern: MVCA provides the enriched context, and FiLM acts as a modulation mechanism that refines these multi-view-augmented features.

3. Effect of Cross-View Attention

Adding cross-view attention on top of MVCA also improves results across all three datasets, raising mean F1 from 62.00% to 64.86% on B-pillar, from 39.55% to 47.71% on head, and from 46.28% to 52.60% on Combined. The gains are generally moderate, although the head subset shows a larger improvement from cross-view attention than from FiLM (+8.16 vs +3.29 F1 points), suggesting that cross-attention captures fine-grained inter-view correspondences that complement the affine modulation provided by FiLM.

4. Full model

The complete model—combining MVCA + FiLM + Cross-Attn—achieves the best F1-score, Recall, and ROC-AUC across all three datasets, although MVCA+FiLM yields slightly higher Precision on the B-pillar and head subsets, with a mean F1 of 70.68% (B-pillar), 53.09% (head), and 69.84% (Combined).

Overall, Table 3 shows a clear and consistent progression:

1. MVCA contributes the largest single improvement and is the key driver of performance gains.
2. FiLM yields consistent additional gains once multi-view context is present.
3. Cross-view attention provides fine-grained fusion that further stabilizes performance.
4. The full model consistently ranks best in F1-score, Recall, and ROC-AUC, especially on the Combined dataset, confirming that MVCA-AD excels under cross-view heterogeneity.

These findings directly confirm the necessity of each component and highlight that Multi-View Context Augmentation is the foundational source of improvement in MVCA-AD.

### 4.4. Interpretability Analysis

**Figure 8:**
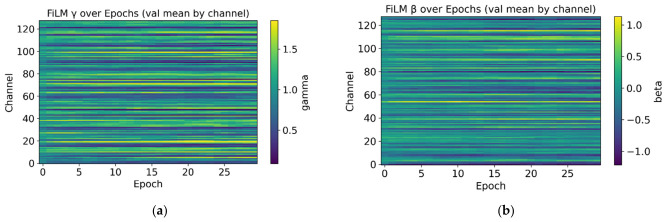
Visualization of the FiLM modulation parameters: (**a**) heatmap of γ across epochs, (**b**) heatmap of β across epochs.

**Figure 9:**
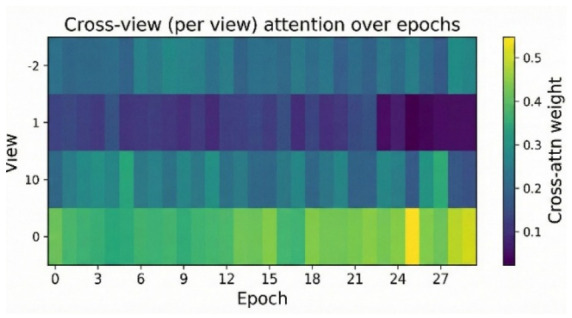
Visualization of the cross-view attention dynamics—heatmap of per-view attention weights across epochs.

To gain further insight into how MVCA-AD integrates multi-view information and adapts its internal representations, we analyze the evolution of FiLM modulation parameters and cross-view attention weights during training. The interpretability results in Figure 8 and Figure 9 highlight distinct and structured learning behaviors for the two modules.

FiLM’s affine parameters (γ and β), visualized as channel-epoch heatmaps in Figure 8, show that modulation is applied independently to each feature channel. A clear pattern emerges: although γ and β differ across channels, both parameters stabilize rapidly with respect to training time. Quantitatively, the majority of channels exhibit minimal temporal fluctuation after approximately five epochs, indicating that FiLM converges early to a consistent modulation profile that selectively scales or shifts features derived from multi-view augmentation. This early stabilization suggests that FiLM primarily learns how to modulate features rather than continuously adapting this behavior throughout training.

In contrast, the cross-view attention heatmap in Figure 9 represents per-view attention weights averaged across all time steps. Unlike FiLM’s per-channel modulation, the cross-view attention tracks how importance is distributed across different view indices (e.g., anchor view, shifted views, derivative views). The attention weights initially vary across epochs as the model explores inter-view dependencies, but gradually settle into a structured, view-differentiated pattern. The curves converge much more slowly than FiLM: attention weights become largely stable after approximately 25 epochs, suggesting that the model refines temporal correspondence and inter-view relevance progressively as feature representations mature.

Overall, these findings show that MVCA-AD does not combine views arbitrarily. FiLM quickly establishes stable per-channel scaling and shifting rules within the first few epochs, while cross-view attention undergoes a longer refinement process before converging to steady importance weights. Together, the two mechanisms form a coherent and interpretable fusion strategy: early channel-wise modulation followed by progressive alignment across complementary views.

## 5. Conclusions

This work proposes the MVCA-AD framework for anomaly detection in single-channel acceleration signals from automotive crash tests. By employing multi-view time–frequency transformations, trend-aware modulation, and cross-view attention fusion, MVCA-AD enables sensitive modeling of multi-scale anomaly patterns.

The contributions of this work can be organized along three complementary dimensions, each corresponding to a limitation of prior methods identified in Section 1. (1) Methodological innovation. To address anomaly scarcity and insufficient feature diversity in conventional single-channel anomaly detection, we introduce a deterministic Multi-View Context Augmentation mechanism that constructs physically interpretable representations across the time, derivative, integral, and time–frequency domains, thereby enriching the feature space without requiring additional anomaly examples. (2) Architectural innovation. To improve robustness against distribution shifts across vehicle models and sensor types, we design a trend-conditioned FiLM modulation block coupled with a multi-head cross-view attention fusion module, which supports scale-aware feature modulation and fine-grained inter-view information exchange while remaining lightweight and end-to-end trainable. (3) Engineering innovation. To make these advances applicable to industrial crash-test data quality control, MVCA-AD operates per channel and is compatible with SAE J211-compliant signals without requiring multichannel synchronization. Its evaluation on physical full-scale crash-test data under event-level grouped splitting across heterogeneous head and B-pillar sensor subsets demonstrates its practical relevance for automated anomalous-curve detection in original equipment manufacturer (OEM) and certification-lab quality-assurance scenarios.

While these results are encouraging, several limitations of the present framework should be acknowledged. First, the current evaluation is conducted on data from a single testing facility under a single crash configuration (full-width rigid barrier); generalization across different laboratories, vehicle platforms, crash configurations, or sensor systems remains to be validated in future work. Second, the framework is optimized for whole-curve binary anomaly detection and may exhibit reduced sensitivity to extremely short anomalous pulses (sub-millisecond transients) that occupy only a small fraction of the analysis window. Third, robustness under extremely high acquisition noise—beyond the typical noise floor of SAE J211-compliant data-acquisition (DAQ) chains—has not been systematically characterized in this study and would require dedicated stress testing under degraded sensor conditions.

In future work, we plan to explore self-supervised learning techniques to improve adaptability to unlabeled data, incorporate graph-based modeling to capture temporal and spatial dependencies across multiple sensors, and extend the framework to other engineering anomaly detection tasks such as structural health monitoring and industrial fault diagnosis, enhancing its generality and scalability.

## Figures and Tables

**Table 1:** Deterministic view library ($T_{k}$) for multi-view expansion.

ID	$\mathrm{View}\;\mathrm{Transformation}\;T_{k}$	Domain	Key Parameters
0	Identity (Anchor)	Time	---
−1	First derivative (central)	Time	---
−2	Second derivative	Time	---
1	First integral	Time	---
2	Second integral	Time	---
10	Fourier filter-bank magnitude	Frequency	bands = 12; kernel = 20 ms; smooth = 5 ms
11	Morlet CWT magnitude	Time–Frequency	scales = 16; cycles = 6
21	2nd-order Butterworth low-pass	Time	cutoff = 60 Hz; order = 2; zero-phase = true

**Table 2:** Performance comparison between MVCA-AD and baselines on three experimental subsets (mean ± standard deviation over 5 grouped splits). Best results are highlighted in bold.

Method	B-Pillar Acc				Head Acc				Head Acc + B-Pillar Acc			
	P	R	F1	AUC	P	R	F1	AUC	P	R	F1	AUC
ROCKET	36.03 ± 0.96	43.63 ± 2.00	39.45 ± 1.03	73.30 ± 1.09	29.58 ± 2.16	40.00 ± 4.49	33.88 ± 2.06	48.84 ± 0.90	32.48 ± 0.88	31.87 ± 2.00	32.15 ± 1.27	59.15 ± 1.16
CNN	0.00 ± 0.00	0.00 ± 0.00	0.00 ± 0.00	64.20 ± 1.71	38.75 ± 4.03	29.74 ± 5.57	33.50 ± 4.82	44.97 ± 2.01	34.66 ± 2.98	6.06 ± 1.35	10.26 ± 2.00	57.38 ± 1.53
FCN	13.00 ± 12.04	3.79 ± 4.32	5.57 ± 5.79	61.58 ± 0.71	33.33 ± 0.00	26.26 ± 5.64	29.11 ± 3.51	43.07 ± 1.25	55.65 ± 3.64	14.14 ± 1.71	22.46 ± 1.98	55.44 ± 1.27
ResNet	0.00 ± 0.00	0.00 ± 0.00	0.00 ± 0.00	36.46 ± 1.24	23.68 ± 1.26	20.14 ± 2.60	21.64 ± 1.26	54.14 ± 1.68	0.00 ± 0.00	0.00 ± 0.00	0.00 ± 0.00	44.42 ± 1.45
DRN	33.47 ± 0.96	57.91 ± 2.48	42.38 ± 0.43	45.45 ± 2.22	29.09 ± 3.00	21.39 ± 3.19	24.44 ± 2.11	52.02 ± 1.44	30.92 ± 0.77	23.02 ± 0.59	26.38 ± 0.31	49.14 ± 1.60
InceptionTime	**81.43 ± 4.24**	47.41 ± 1.57	59.89 ± 1.87	86.60 ± 1.11	**50.67 ± 1.49**	53.88 ± 4.19	52.13 ± 1.66	48.88 ± 1.04	51.72 ± 1.59	31.74 ± 0.64	39.33 ± 0.64	64.88 ± 0.81
TCN	35.00 ± 9.13	7.00 ± 1.69	11.50 ± 2.32	41.28 ± 1.54	17.34 ± 1.49	16.38 ± 3.45	16.73 ± 2.43	53.31 ± 1.94	40.00 ± 22.36	2.63 ± 1.51	4.93 ± 2.83	47.24 ± 1.97
LSTM	44.96 ± 5.27	15.20 ± 0.89	22.69 ± 1.57	47.23 ± 1.77	25.98 ± 0.98	31.27 ± 6.84	28.12 ± 3.48	48.41 ± 0.97	22.66 ± 0.33	23.02 ± 0.59	22.84 ± 0.40	45.21 ± 0.98
GRU	41.04 ± 1.83	36.62 ± 1.37	38.69 ± 1.22	61.67 ± 1.21	25.49 ± 2.35	28.73 ± 7.69	26.71 ± 3.91	48.32 ± 0.66	25.51 ± 0.34	43.90 ± 1.30	32.26 ± 0.54	51.37 ± 0.55
MVCA-AD	61.06 ± 0.63	**83.90 ± 1.18**	**70.68 ± 0.81**	**90.07 ± 1.66**	46.48 ± 2.32	**62.23 ± 4.49**	**53.09 ± 1.52**	**79.86 ± 1.14**	**59.57 ± 0.67**	**84.41 ± 0.79**	**69.84 ± 0.34**	**84.46 ± 1.43**

**Table 3:** Ablation study on key components (mean ± standard deviation over 5 grouped splits). Best results are highlighted in bold.

MVCA	FiLM	Cross-Attn	B-Pillar Acc				Head Acc				Head Acc + B-Pillar Acc			
			P	R	F1	AUC	P	R	F1	AUC	P	R	F1	AUC
			33.47 ± 0.96	57.91 ± 2.48	42.38 ± 0.43	45.45 ± 2.22	29.09 ± 3.00	21.39 ± 3.19	24.44 ± 2.11	52.02 ± 1.44	30.92 ± 0.77	23.02 ± 0.59	26.38 ± 0.31	49.14 ± 1.60
√			57.05 ± 1.71	67.93 ± 1.48	62.00 ± 1.37	85.94 ± 1.34	38.14 ± 1.75	41.53 ± 5.11	39.55 ± 1.88	68.20 ± 1.76	43.67 ± 0.55	49.27 ± 2.42	46.28 ± 1.18	72.40 ± 1.10
	√		39.44 ± 0.87	52.73 ± 4.10	45.04 ± 1.17	82.71 ± 1.93	29.98 ± 1.97	30.40 ± 8.52	29.41 ± 4.26	59.21 ± 1.85	37.36 ± 0.54	33.73 ± 1.53	35.43 ± 0.74	67.56 ± 1.87
√	√		**63.42 ± 0.57**	74.02 ± 1.67	68.30 ± 0.41	88.46 ± 0.98	**48.00 ± 4.47**	38.75 ± 4.03	42.84 ± 3.96	74.75 ± 1.84	57.47 ± 1.01	67.49 ± 1.23	62.06 ± 0.61	79.25 ± 1.88
√		√	58.18 ± 1.22	73.39 ± 2.86	64.86 ± 0.48	87.57 ± 0.74	44.08 ± 3.84	52.34 ± 5.44	47.71 ± 3.29	72.50 ± 1.79	48.55 ± 0.21	57.39 ± 0.86	52.60 ± 0.45	77.77 ± 2.05
√	√	√	61.06 ± 0.63	**83.90 ± 1.18**	**70.68 ± 0.81**	**90.07 ± 1.66**	46.48 ± 2.32	**62.23 ± 4.49**	**53.09 ± 1.52**	**79.86 ± 1.14**	**59.57 ± 0.67**	**84.41 ± 0.79**	**69.84 ± 0.34**	**84.46 ± 1.43**

## Data Availability

The vehicle crash-test time-series data used in this study are not publicly available due to restrictions imposed by the data provider, but detailed descriptions of the data are provided in the paper. The results generated in this study are available within the paper.

## Abbreviations

The following abbreviations are used in this manuscript:

MVCA-AD	Multi-View Context Augmentation for Anomaly Detection
MVCA	Multi-View Context Augmentation
FiLM	Feature-wise Linear Modulation
AE	AutoEncoder
VAE	Variational AutoEncoder
LSTM	Long Short-Term Memory
GRU	Gated Recurrent Unit
TCN	Temporal Convolutional Network
SVDD	Support Vector Data Description
DRN	Dilated Residual Network
CWT	Continuous Wavelet Transform
CNN	Convolutional Neural Network
FCN	Fully Convolutional Network
MLP	Multilayer Perceptron
FFN	Feedforward Network
ReLU	Rectified Linear Unit
GELU	Gaussian Error Linear Unit
PPV	Proportion of Positive Values
ROC-AUC	Area Under the Receiver Operating Characteristic Curve
